# High Physiological Omega-3 Fatty Acid Supplementation Affects Muscle Fatty Acid Composition and Glucose and Insulin Homeostasis in Obese Adolescents

**DOI:** 10.1155/2012/395757

**Published:** 2012-02-20

**Authors:** Frida Dangardt, Yun Chen, Eva Gronowitz, Jovanna Dahlgren, Peter Friberg, Birgitta Strandvik

**Affiliations:** ^1^Department of Molecular and Clinical Medicine, Clinical Physiology, Sahlgrenska Academy at the University of Gothenburg, 416 85 Gothenburg, Sweden; ^2^Department of Paediatrics, Sahlgrenska Academy at the University of Gothenburg, 416 85 Gothenburg, Sweden; ^3^Unit of Public Health Nutrition, Department of Biosciences and Nutrition, Novum at Karolinska Institute, 141 86 Stockholm, Sweden

## Abstract

Obese adolescents have high concentrations of saturated fatty acids and low omega-3 long-chain polyunsaturated fatty acids (LCUFAs) in plasma phospholipids. We aimed to investigate effects of omega-3 LCPUFA supplementation to obese adolescents on skeletal muscle lipids and glucose and insulin homeostasis. Twenty-five obese adolescents (14–17 years old, 14 females) completed a randomized double-blind crossover study supplying capsules containing either 1.2 g omega-3 LCPUFAs or placebo, for 3 months each with a six-week washout period. Fasting blood glucose, insulin, leptin, adiponectin, and lipids were measured. Intravenous glucose tolerance test (IVGTT) and euglycemic-hyperinsulinemic clamp were performed, and skeletal muscle biopsies were obtained at the end of each period. The concentrations of EPA, DHA, and total omega-3 PUFA in muscle phospholipids increased in both sexes. In the females, omega-3 LCPUFA supplementation improved glucose tolerance by 39% (*P* = 0.04) and restored insulin concentration by 34% (*P* = 0.02) during IVGTT. Insulin sensitivity improved 17% (*P* = 0.07). In males, none of these parameters was influenced by omega-3 supplementation. Thus, three months of supplementation of omega-3 LCPUFA improved glucose and insulin homeostasis in obese girls without influencing body weight.

## 1. Introduction

The prevalence of childhood obesity continues to increase [1], and obesity-related adverse conditions, such as high blood pressure, increased left ventricular mass, insulin resistance, and type 2 diabetes, are now being seen in children with increasing frequency [2, 3]. The association of obesity with type 2 diabetes has been recognized for decades, and the major basis for this link is the characteristic association of obesity to insulin resistance. Insulin resistance plays an important role in the pathogenesis of the metabolic syndrome, and yet, the mechanisms remain enigmatic.

It has been shown recently that obese children, with their prevailing risk profile, have lower serum levels of the essential fatty acids (FAs) of the omega-3 series than age-matched lean controls [4, 5]. Risk factors for cardiovascular disease have mainly been referred to saturated FA and trans FA, but diabetes and obesity have also been suggested to more relate to decreased omega-3 FA than to the total amount of dietary fat [6–8]. Earlier studies have demonstrated that increasing the omega-3 LCPUFA concentrations, using Mediterranean inspired diet or omega-3 supplementation, lowered plasma triacylglycerols, inflammatory markers, platelets, and white blood cells in both healthy and obese individuals [9–11]. Omega-3 LCPUFA supplementation has also been shown to improve insulin sensitivity in overweight adults [12].

The skeletal muscle, liver, and adipose tissue are known to play a pivotal role in the development of insulin resistance [13, 14]. Insulin sensitivity has been shown to be related to the FA composition of muscle phospholipids [15]. Hence, we hypothesized that increasing the omega-3 LCPUFA levels in the skeletal muscle might have beneficial influence on metabolism and cardiovascular risk in obese adolescents. The purpose of the present study was to investigate the effect of omega-3 LCPUFA supplementation on glucose and insulin metabolism in obese adolescents more thoroughly. Given that girls with obesity are at greater risk for impaired insulin sensitivity than boys [16], analyses of data were separated by gender. In order to focus on the effect of the supplementation, no intervention was made for physical activity or diet.

## 2. Subjects and Methods

### 2.1. Study Subjects

One-hundred and eight obese adolescents (14–17 years), referred to our outpatient clinic, were invited to enroll in the study. Only 47 participated in an information meeting, and 31 of these agreed to participate. One subject was excluded due to smoking, one moved abroad, three withdrawn during the study, and one was excluded due to poor compliance. Thus, 14 females (aged 15.6 ± 0.9, BMI z-score 2.1 ± 0.2) and 11 males (aged 15.7 ± 1.0, BMI z-score 2.4 ± 0.4) completed the study. For anthropometry, see Table 1. Information and written protocols, approved by the Ethics Committee of the Sahlgrenska Academy at the University of Gothenburg, were presented, and written consent was obtained from the adolescents and their parents. Obesity was defined by the International Obesity Task Force criteria [17]. No adolescent reported chronic disease or regular use of medications. All were at pubertal Tanner stage > 4 regarding both gonad size and pubic hair. All females were after menarche.

### 2.2. Study Design

The study was performed in a randomized double-blind, crossover design with a six-week washout period. A random number generator performed the randomization. Each adolescent was randomized to a daily intake of 10 capsules (EyeQ, Equazen, Wigan, UK), providing 1.2 g omega-3 LCPUFA (930 mg eicosapentaenoic acid (EPA, 20 : 5n-3), 290 mg docosahexaenoic acid (DHA, 22 : 6n-3), 100 mg *γ*-linolenic acid (GLA, 18 : 3n-6), and 18 mg vitamin E daily or placebo capsules containing medium chain triglycerides (MCT); in total the same amount of fats was given with both treatments. The subjects were instructed to take the capsules once a day before breakfast. Physical examination, blood samples, and anthropometric measurements were taken before the supplementation and after each 3-month period. Serum phospholipid FA analyses were performed at the end of the washout period to verify that the omega-3 LCPUFA concentrations were back to baseline levels.

At the end of each period, muscle biopsies were obtained, and intravenous glucose tolerance test (IVGTT) and hyperinsulinemic-euglycemic clamp were performed. Results of investigation of vascular function and inflammation have previously been reported [18].

The adherence to the supplementation was monitored by monthly contacts. Leftover capsules and records of capsule intake were presented to one of the investigators, and one subject with compliance below 75% was excluded. For control of the diet, subjects completed a semiquantitative food-frequency questionnaire before each period, which revealed no difference in dietary intake pattern. For the physical activity, the subjects were asked to keep their habits during the study, and according to the regular checkups done during the study periods, there was no change in the physical activity.

### 2.3. Intravenous Glucose Tolerance Test (IVGTT)

After an overnight fast, two catheters were inserted, one into the left antecubital vein for infusions of glucose, and another one into an ipsilateral heated dorsal hand vein for withdrawal of arterialized venous blood. After baseline blood collection, 300 mg glucose/kg body weight (30% glucose solution) was given within two minutes. Blood samples were drawn every second minute to 10 minutes, thereafter every 5 minutes to 30 minutes and then every 10 minutes to 120 minutes after the glucose infusion. Plasma glucose was analyzed with a glucose analyzer (HemoCue Glucose 201 DM Analyzer, HemoCue AB, Sweden), and serum insulin was analyzed by Enzyme-Linked Immunosorbent Assay (ELISA, Mercodia, Uppsala, Sweden). Analysis of glucose and insulin response to IVGTT was performed according to Kahn et al. [19]. The magnitude of insulin response to glucose was quantified as the incremental area under the curve (ΔAUC) and divided into different time periods, 0–10 minutes and 60–80 minutes, reflecting the first phase insulin secretion and the restoration of insulin concentration, respectively. Glucose disappearance constant (Kg) was calculated as the slope of the logarithm of glucose values between 10 and 30 minutes after the glucose infusion.

### 2.4. Euglycemic Hyperinsulinemic Clamp

Thirty minutes after the IVGTT, a euglycemic hyperinsulinemic clamp was carried out for evaluating insulin sensitivity. This design has been previously validated [20]. Human insulin (Actrapid, Novo Nordisk, Copenhagen, Denmark) was infused as a priming dose, followed by continuous infusion (80 mU/m^2^ body surface/min) for 120 minutes. Glucose infusion was started simultaneously and the infusion rate adjusted to clamp the blood glucose at 5.0 mmol/L, assessed at 5-minute intervals using the glucose analyzer. The glucose infusion rate during the last 60 minutes was a measure of the subject's insulin sensitivity expressed as glucose disposal rate (GDR) (mg/kg body weight/min). The GDR (M-value) was calculated as the mean value of the glucose infused for each 20-minute interval during the last 60 minutes of the clamp. The insulin sensitivity index (ISI) was calculated by dividing the M-value by the steady-state insulin concentration during the last 60 minutes of the clamp (mg glucose/kg body weight/min/insulin (mU/L)) [20].

### 2.5. Blood Sample Analysis

Fasting blood samples were taken before and after the supplementations. Blood glucose, total cholesterol, HDL-cholesterol, LDL-cholesterol, and triacylglycerols were analyzed by enzymatic methods (Roche Diagnostics, Mannheim, Germany) according to routine (Sahlgrenska University Hospital, Göteborg). Fasting serum insulin was analysed with ELISA. The intra-assay coefficient of variation for insulin was 3.3%. Plasma leptin was analyzed by ELISA from Mercodia (Uppsala, Sweden) and high-molecular weight-adiponectin (HMW-Adiponectin) was analyzed by ELISA from Linco (St. Charles, Missouri, USA). Coefficients of variation within assays were 3.7% for leptin and 16% for HMW-Adiponectin. Some of the results have previously been reported for the whole study sample but are here given by gender [18].

### 2.6. Biopsies

After intra- and subcutaneous administration of local anaesthesia (Mepivacain, 10 mg/mL, Carbocain, Astrazeneca, Sweden), muscle biopsies were obtained from M. vastus lateralis with a Bergmann needle after each period from 18 (11 female) of the subjects. Biopsies were immediately frozen in liquid nitrogen and stored at −70°C until analysis.

### 2.7. Fatty Acid Analysis

Fatty acid analyses were performed in serum and muscle biopsies. After lipid extraction, serum phospholipids were fractionated on a single SEP-PAK aminopropyl cartridge (Waters Corp., Beverly, MA, USA), transmethylated, and separated by capillary GLC in a Hewlett-Packard 6890 gas chromatograph (Palo Alto, CA, USA) as previously reported [21]. The biopsies were homogenized with a Polytron PT 1200 homogenizer (Kinematica AG) in 400 *μ*L of water. After the addition of isopropanol, the homogenate was sonicated and chloroform was added, together with 100 *μ*g n-oleoyl-ethanolamine as internal standard (Sigma-Aldrich, Sweden AB, Stockholm) and 2,6-di-tert-butyl-4-methylphenol (BHT) 0.1 mg/mL chloroform. The homogenate was incubated for 1 hour and then centrifuged at 2000 g for 10 minutes. After extraction with chloroform-methanol solution (2 : 1), the extract was filtered (Acrodisc PTFE, 0.45-*μ*m filtering capacity; Waters Corporation, Milford, MA). The filtrate was evaporated and resolved in 100 *μ*L hexane: 2-propanol: HAc: 3-ethylamine solution (50 : 50 : 1.5 : 0.1). The sample was injected in a high performance liquid chromatograph with a LiChrospher 100 DIOL column (dimension = 250 × 4 mm; film thickness = 5 *μ*m) at 55°C with split flow to an evaporative light-scattering detector and fraction collection. Hexane: 2-propanol: HAc: 3-ethylamine (82 : 17 : 1.5 : 0.1) and 2-propanol: H2O: HAc: 3-ethylamine (84 : 15 : 1.5 : 0.1) solutions were used as binary gradients. The flow was 0.8 mL/min. Fractions were collected for phosphatidylcholine, phosphatidylethanolamine, phosphatidylserine, phosphatidylinositol, diphosphoglycerides, phosphoglycerides, and sphingomyelin. The two latter were small, and those and the neutral lipid fraction were not further analysed, but different phospholipid fractions were esterified by incubation for 4 hours at a temperature of 80°C with methanol HCl-3 N and 2 *μ*g heneicosanoic acid methyl ester added as an internal standard. The methyl esters were extracted with hexane, washed with Millipore water and dried over MgSO_4_ and analysed on GLC as previously described [21].

### 2.8. Statistical Analysis

Statistical analyses were performed with the SPSS 15.0. Paired samples *t*-test was used where applicable. Variables that were not normally distributed were analyzed by Wilcoxon signed-ranks test. All results are expressed as means ± SD or median and range where applicable. GraphPad Prism 4.03 was used for all curve analysis of glucose and insulin response during IVGTT, specifically area under curve and slope. A *P* < 0.05 was considered statistically significant.

## 3. Results

The baseline parameters of the omega-3 LCPUFA supplementation periods did not differ from those of the placebo periods (Table 1). No significant differences between the omega-3 LCPUFA and the placebo supplementations were found regarding body weight, BMI, or any blood parameters (Table 1). The omega-3 LCPUFA supplementation significantly increased the omega-3 LCPUFA concentrations in phospholipids of serum (Table 1, [18]) and skeletal muscle (Table 2), as compared with placebo supplementation.

### 3.1. Glucose Tolerance and Insulin Sensitivity

**Figure 1:**
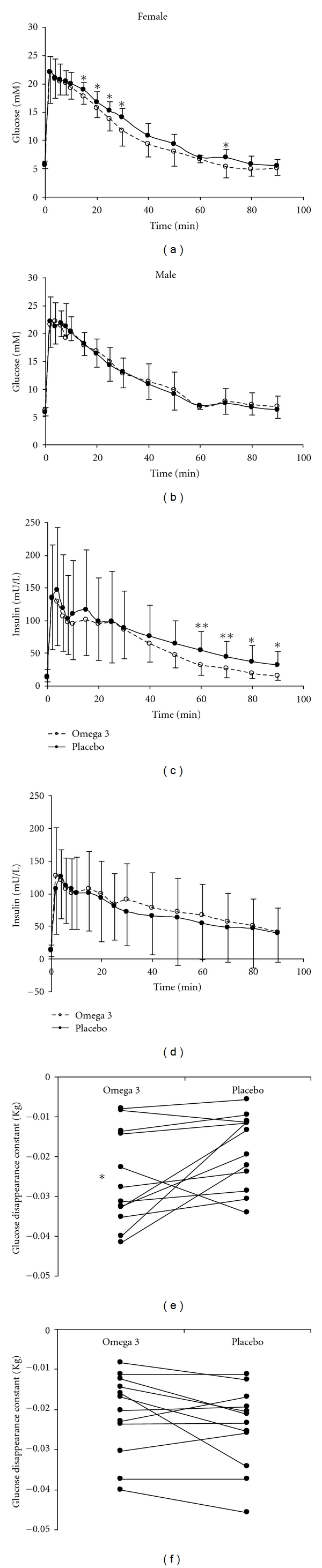
Intravenous glucose tolerance test: glucose (upper panels) and insulin (middle panels) response curves after omega-3 LCPUFA or placebo supplementation, in male (right panels) and female (left panels) obese adolescents. Omega-3 LCPUFA supplementation increased glucose disappearance rate (lower panels) and improved restoration of insulin concentration in female, but not male subjects.*P < 0.05, **P < 0.01 compared with placebo.

In females, the glucose tolerance (Kg) was significantly improved after omega-3 LCPUFA supplementation (omega-3: −0.0257 ± 0.0119; control: −0.0185 ± 0.0093, *P* < 0.05, Figure 1). In line with this, ISI obtained from euglycemic-hyperinsulinemic clamp tended to be increased (omega-3: 0.047 ± 0.016; control: 0.039 ± 0.015 mg·kg^−1^·min^−1^·(mU/L)^−1^, *P* = 0.07). The restoration of insulin concentration during IVGTT was also improved (ΔAUC_60−80 min⁡_  , omega-3:  616 ± 425  and placebo:  937 ± 496  mU·L^−1^·min^−1^, *P* = 0.02, Figure 1). The omega-3 LCPUFA supplementation did not change the first-phase insulin secretion during IVGTT (ΔAUC_0−10 min⁡_) (omega-3: 1098 ± 559; placebo:  1174 ± 726  mU·L^−1^·min^−1^) or GDR obtained from euglycemic-hyperinsulinemic clamp (omega-3: 6.8 ± 3.7, placebo: 7.4 ± 3.2 mg/kg body weight/min).

In males, the omega-3 LCPUFA supplementation did not change the Kg (omega-3: −0.022 ± 0.0103; placebo: −0.025 ± 0.0106, Figure 1), the ISI (omga-3: 0.046 ± 0.034 and placebo: 0.051 ± 0.041 mg·kg^−1^·min^−1^· (mU/L)^−1^), the GDR (omega-3: 6.8 ± 3.7; placebo: 7.4 ± 3.2 mg/kg body weight/min), the restoration of insulin concentration (ΔAUC_60–80 min⁡_, omega-3:  1390 ± 1026  and placebo: 1002 ± 1092, Figure 1), or the ΔAUC_0–10 min⁡_ (omega-3: 1076 ± 400; control:  1050 ± 552  mU·L^−1^·min^−1^).

### 3.2. Muscle Fatty Acid Composition

In skeletal muscle phospholipids (Table 2), the percentage of total omega-3 PUFAs was 47% and 45% higher after omega-3 LCPUFA supplementation than after placebo in females and males, respectively. The percentage of total omega-6 PUFAs and linoleic acid (LA, 18:2n-6) decreased in both females and males, while arachidonic acid (AA, 20 : 4n-6) decreased only in males (Table 2). The concentration of EPA was increased more than 115% after omega-3 LCPUFA supplementation in both females and males. Significant increase in DHA was also seen. Accordingly, the omega-6/omega-3 ratio was lower with omega-3 LCPUFA supplementation than with placebo. Total or individual SFA and MUFA were not changed. Similar pattern of FA changes were found in phosphatidylethanolamine and phosphatidylcholine, whereas in phosphatidylserine omega-6 FA was lower with the omega-3 supplementation. The FA composition of phosphatidylinositol and diphosphoglycerides (cardiolipin) remained largely unchanged.

The triacylglycerols in muscles were lower after omega-3 supplementation than after placebo (females omega-3 : 25.5 (9.5–116.0), placebo: 38.7 (7.8–86.1) mg/g wet weight and males omega-3 : 39.4 (11.7–57.3) and placebo: 46.2 (26.6–140.0) mg/g wet weight). However, the differences were not significant.

The placebo capsules did not cause any significant changes in serum concentrations of glucose, insulin and triacylglycerols (Table 1), suggesting that the MCT did not affect the insulin-glucose metabolism and fatty acid oxidation. The absence of any influence on body weight also indicated that the energy supply was negligible. Carryover effects of receiving omega-3 LCPUFA or MCT capsules first were checked for the analysed variables, and no such effects were found.

## 4. Discussion

This study shows that omega-3 LCPUFA supplementation in a high physiological dose of 1.2 g/day for 3 months improved glucose and insulin homeostasis in obese girls without concomitant weight reduction. This effect was not found in the obese boys. The omega-3 LCPUFA supplementation also increased incorporation of omega-3 LCPUFAs into the skeletal muscle phospholipids in both gender, as reflected in the increased levels of omega-3 LCPUFAs in this tissue. The triacylglycerides in muscle were decreased, although not reaching significance.

Glucose tolerance, determined as glucose disappearance constant (Kg) during IVGTT, is influenced by insulin secretion from the pancreatic *β*-cells and insulin sensitivity in the liver and peripheral tissues [22]. The increased glucose tolerance after the omega-3 LCPUFA supplementation in females was probably due to an improvement in insulin sensitivity rather than insulin secretion, since the first-phase of insulin secretion was not changed by the omega-3 LCPUFA supplementation. In line with this, we found a moderate increase of insulin sensitivity, determined as ISI by the euglycemic-hyperinsulinemic clamp. Thus, our data indicate that omega-3 LCUFAs, or the balance between omega-6 and omega-3 fatty acids, play a role in the insulin and glucose metabolism in obese girls at this young age. The differences induced by the omega-3 LCPUFA supplementation were often slightly higher in the females, reflected in a higher significance level, which might indicate that boys might need a longer or more intensive supplementation. The difference in results might also be related to the lower number of boys in the study.

Glucose tolerance and peripheral insulin sensitivity are mostly regulated by the FA composition in the liver and skeletal muscle [7, 23]. It has been shown that obese children with impaired glucose tolerance demonstrate defects in nonoxidative glucose metabolism, and intramyocellular lipid accumulation is related to insulin resistance [13]. A number of different metabolic abnormalities may increase intramyocellular FA metabolites, such as increased fat delivery to muscle as a consequence of either excess energy intake, liver disturbances, or defects in adipocyte fat metabolism [24]. Another possible mechanism behind intramyocellular lipid accumulation would be reduced mitochondrial oxidative phosphorylation, since mitochondria within the cells convert FA and glucose into energy via beta-oxidation. Indeed, mitochondrial dysfunction causes deposits of lipids inside the muscle, producing insulin resistance and leading to diabetes [25]. It is possible that the improved glucose and insulin metabolism after omega-3 LCPUFA supplementation can be attributed to decreased muscle triacylglycerides. We found that omega-3 LCPUFA supplementation led to a nonsignificant decrease in the level of muscle triacylglycerides (34% in girls and 15% in boys), which might support such hypothesis. Decreased insulin sensitivity has been shown to be associated with decreased total percentage of C20–22 PUFAs in skeletal muscle phospholipids [15]. In our studies, the omega-3 LCPUFA supplementation increased total percentage of C20–22 PUFAs of the omega-3 series in skeletal muscle phospholipids. Since mitochondria are rich in DHA, it was of interest that in phosphatidylethanolamine and phosphatidylserine, especially concentrated in the inner membrane of the mitochondria [26], there were significant changes in the ratios between long chain omega-6 and omega-3 fatty acids. In phosphatidylethanolamine, there were large changes in the concentration of EPA, DHA, and AA. Our data in obese girls are intriguing inasmuch as the changes in the FA composition of muscles may contribute to the improved glucose and insulin metabolism. A large study completed with analyses of membrane receptors and transcription factors involved in glucose/insulin metabolism would be of interest.

A decrease in visceral fat would also be associated with increased insulin sensitivity [13], but during this relatively short time of the study anthropometry did not change. A long-term study would be of interest, in the context that Huber and co-workers found that a high-fat diet induced adipose tissue remodelling in obese diabetic mice was prevented by supplementation with omega-3 LCPUFA [27].

The omega-3 LCPUFA supplementation improved glucose and insulin metabolism only in the females in spite of favourable changes in skeletal muscle phospholipid FA in both sexes. There were fewer boys, but factors other than changes in muscle FA may be involved in the improved glucose and insulin metabolism induced by omega-3 LCPUFA supplementation. Gender differences in omega-3 PUFA metabolism have been reported (for review, see [28]). Women, but not men, responded to a high oleate/low palmitate diet by markedly increased FA oxidation [29]. Interestingly, Phang demonstrated that gender differences existed in platelet aggregation in response to various omega-3 LCPUFA supplements [30]. It might be possible that dietary FA, such as omega-3 LCPUFAs, and sex hormones (particularly oestrogen) can act synergistically to improve glucose and insulin metabolism in female obese adolescents. This warrants further investigations.

There is evidence that the impaired insulin sensitivity in obese girls starts even before puberty. In a study of healthy 4-year olds, we found that in girls, but not in boys, serum fasting insulin concentration and HOMA-IR were associated with the weight gain from birth to 4 years, and higher weight was found in those with low omega-3 FA intake [31]. Fatty acid composition of food intake, that is, high intake of omega-6 PUFAs, early in life is also related to the development of insulin resistance and obesity in children [32]. High weight gain and symptoms of the metabolic syndrome in adult rats, which only in the perinatal period were given a ratio of essential fatty acids of omega-6/omega-3 of 9 : 1, commonly found in human diets, indicated that programming by FA might occur perinatally resulting in diseases later in life [33]. Insulin resistance was found in rats with n-3 FA deficiency [34], and we have shown earlier that obese children had lower concentrations of omega-3 fatty acids in serum phospholipids [4], which may contribute to the development of insulin resistance.

Major limitations of our study are the small number of subjects and the short time of intervention. Notwithstanding these restraints, we showed that 3 months of intervention with omega-3 LCPUFAs in a high physiological dose was sufficient to improve glucose and insulin metabolism in obese girls, as well as the FA pattern in the skeletal muscle in both gender. Our results are in agreement with a recently published study in obese mice [35]. High doses, such as 4 g/day used in other studies [36], would probably give stronger effects, but are also less physiological considering that the subjects did not have diabetes. Clinical investigations of obese children and adolescents approaching basic physiological effects might provide a more “pure situation” with little influence of other confounding risk factors and would thus be valuable to extend.

In conclusion, after a relatively short intervention period with 1.2 g/day of omega-3 LCPUFA supplementation, the fatty acid composition of skeletal muscle phospholipids and muscle triacylglycerols improved in both obese boys and girls. Improved glucose and insulin metabolism in the obese girls indicated a specific effect since anthropometry did not change. Girls have previously been found to be at higher risk of developing insulin resistance [16], an abnormality that might still be reversible at this early age. Long-term studies in larger group of children would be most important to verify these preliminary results.

## Figures and Tables

**Table 1:** Anthropometric and biochemical measures at baseline and after 3-month treatment with *n*-3 PUFAs or placebo in 11 male and 14 female obese subjects.

	Male					Female				
	Omega 3		Control		*P* value	Omega 3		Control		*P* value
	Baseline	After 3-month treatment	Baseline	After 3-month treatment		Baseline	After 3-month treatment	Baseline	After 3-month treatment	
Height (m)	1.75 ± 0.06	1.77 ± 0.06	1.76 ± 0.06	1.77 ± 0.06	ns	1.65 ± 0.06	1.66 ± 0.06	1.66 ± 0.06	1.67 ± 0.06	0.002
Weight (kg)	108.8 ± 19.4	111.7 ± 20.11	111.0 ± 19.7	111.7 ± 19.3	ns	90.9 ± 11.1	91.8 ± 12.0	91.6 ± 12.4	93.6 ± 13.0	ns
BMI (kg/m2)	35.2 ± 5.1	35.7 ± 5.4	35.7 ± 5.1	35.5 ± 5.0	ns	33.1 ± 2.7	33.1 ± 3.2	33.1 ± 3.4	33.7 ± 3.5	ns
Waist (cm)	113.8 ± 14.1	111.4 ± 13.3	116.8 ± 13.0	111.6 ± 12.5	ns	106.6 ± 9.1	98.4 ± 9.1	104.5 ± 11.8	97.6 ± 9.1	ns
Hip (cm)	116.8 ± 12.0	116.0 ± 12.3	120.5 ± 9.1	118.4 ± 10.5	ns	116.3 ± 7.0	112.5 ± 6.5	116.5 ± 5.5	113.0 ± 6.1	ns
Serum phospholipid w-3 concentration (mol%)	4.9 ± 1.1	8.0 ± 1.5	5.3 ± 1.2	5.1 ± 1.1	<0.0001	5.9 ± 1.5	10.0 ± 2.5	6.4 ± 1.3	6.4 ± 2.6	0.001
Total cholesterol (mmol/L)	4.0 ± 0.6	3.9 ± 0.5	4.2 ± 0.7	3.8 ± 0.7	ns	4.3 ± 0.7	4.1 ± 0.7	4.2 ± 0.7	3.9 ± 0.7	ns
Triacylglycerol (mmol/L)	1.5 ± 0.6	1.1 ± 0.4	1.3 ± 0.5	1.1 ± 0.5	ns	1.2 ± 0.5	0.9 ± 0.4	1.2 ± 0.6	1.1 ± 0.6	ns
HDL cholesterol (mmol/L)	1.2 ± 0.2	1.2 ± 0.2	1.2 ± 0.2	1.2 ± 0.2	ns	1.3 ± 0.3	1.3 ± 0.2	1.3 ± 0.2	1.3 ± 0.2	ns
LDL cholesterol (mmol/L)	2.2 ± 0.6	2.2 ± 0.5	2.4 ± 0.6	2.1 ± 0.6	0.07	2.4 ± 0.6	2.2 ± 0.6	2.3 ± 0.6	2.1 ± 0.7	ns
Glucose (mmol/L)	4.8 ± 0.4	5.2 ± 0.4	4.9 ± 0.5	5.0 ± 0.3	ns	4.4 ± 0.3	4.8 ± 0.3	4.6 ± 0.4	4.7 ± 0.4	ns
Insulin (*μ*U/L)	13.0 ± 6.7	14.4 ± 8.4	14.5 ± 8.9	10.0 ± 3.8	ns	10.6 ± 4.6	12.9 ± 5.7	11.6 ± 4.2	13.9 ± 11.1	ns
HMW-adiponectin (mg/mL)	3.2 ± 1.6	3.0 ± 2.0	4.0 ± 2.4	3.9 ± 2.6	ns	5.5 ± 3.8	5.2 ± 4.5	5.4 ± 2.7	4.3 ± 2.7	ns
Leptin (ng/mL)	27 ± 19	26 ± 18	29 ± 20	29 ± 22	ns	53 ± 18	57 ± 16	53 ± 18	63 ± 23	ns

Data are presented as mean ± SD. Statistical significance was calculated as the difference between the starting value and that at the end of the treatment period for each treatment arm, and these differences were tested by paired *t* test or Wilcoxon signed-ranks test for nonnormally distributed variables. For all variables, there were no significant differences between the means at the beginning of the 2 treatment periods. BMI, body mass index; HMW, high molecular weight.

**Table 2:** Fatty acid compositions of total muscle phospholipids and of major glycerophospholipids, phosphatidylcholine (PC), phosphatidylethanolamine (PE), phosphatidylserine (PS), phosphatidylinositol (PI), and cardiolipin (DPG) in 12 females and 7 males after 3-months treatment with omega-3 LCPUFAs or placebo, expressed as molar percentage of total FA.

	Female (*n* = 12)		*P* value	Male (*n* = 7)		*P* value
	*ω*-3	Control		*ω*-3	Control	
*Σ*SFA	32.6 ± 1.5	33.2 ± 1.5	ns	31.6 ± 0.4	32.0 ± 0.6	ns
*Σ*MUFA	6.8 ± 1.0	7.1 ± 0.9	ns	6.0 ± 0.8	6.4 ± 1.0	ns
*Σ*PUFA	60.6 ± 1.8	59.7 ± 2.0	ns	62.3 ± 1.0	61.6 ± 1.4	ns
*Σ*ω*-6*	52.8 ± 2.4	54.4 ± 2.5	0.019	53.8 ± 1.8	56.0 ± 1.5	0.013
*18 : 2*ω*-6 (LA)*	38.7 ± 2.4	40.1 ± 2.2	0.017	38.9 ± 1.8	40.3 ± 1.9	0.047
*20 : 4*ω*-6 *(AA)	12.1 ± 1.2	12.1 ± 1.3	ns	12.7 ± 1.8	13.5 ± 1.8	0.027
*Σ*ω*-3*	7.5 ± 0.9	5.1 ± 1.1	<0.0001	7.7 ± 1.8	5.3 ± 1.7	<0.0001
*18 : 3*ω*-3 *(ALA)	0.4 ± 0.1	0.4 ± 0.1	ns	0.3 ± 0.0	0.4 ± 0.1	ns
*20 : 5*ω*-3 *(EPA)	2.5 ± 0.5	1.1 ± 0.4	<0.0001	2.8 ± 0.6	1.3 ± 0.6	<0.0001
*22 : 6*ω*-3 *(DHA)	2.8 ± 0.4	2.1 ± 0.6	<0.0001	2.8 ± 1.0	2.1 ± 0.9	0.001
**ω*-6/*ω*-3 *ratio	7.1 ± 1.2	11.3 ± 2.8	<0.0001	7.3 ± 2.0	11.7 ± 3.9	0.002

PC						
*Σ*ω*-6*	42.5 ± 4.9	45.2 ± 1.6	0.026	45.0 ± 0.7	45.1 ± 1.6	ns
*18 : 2*ω*-6 *(LA)	39.0 ± 4.8	41.5 ± 1.7	0.091	41.0 ± 1.2	41.2 ± 2.0	ns
*20 : 4*ω*-6 *(AA)	2.4 ± 0.2	2.5 ± 0.3	ns	2.8 ± 0.5	2.8 ± 0.4	ns
*Σ*ω*-3*	2.4 ± 0.6	1.6 ± 0.5	0.003	2.4 ± 0.5	1.7 ± 0.6	0.018
*18 : 3*ω*-3 *(ALA)	0.4 ± 0.2	0.4 ± 0.2	ns	0.4 ± 0.0	0.4 ± 0.1	ns
*20 : 5*ω*-3 *(EPA)	0.8 ± 0.2	0.4 ± 0.2	0.003	0.9 ± 0.3	0.4 ± 0.2	0.018
*22 : 6*ω*-3 *(DHA)	0.6 ± 0.3	0.4 ± 0.1	0.012	0.6 ± 0.2	0.4 ± 0.2	0.089
**ω*-6/*ω*-3 *ratio	19.0 ± 5.0	31.0 ± 9.3	0.003	20.0 ± 5.2	30.0 ± 11.0	0.018

PE						
*Σ*ω*-6*	37.9 ± 4.2	43.7 ± 2.4	0.005	39.0 ± 4.0	43.4 ± 4.7	0.028
*18 : 2*ω*-6 *(LA)	10.1 ± 1.9	11.4 ± 1.4	0.037	9.8 ± 2.6	10.4 ± 2.8	0.075
*20 : 4*ω*-6 *(AA)	27.4 ± 3.4	31.8 ± 3.3	0.005	28.6 ± 3.1	32.4 ± 4.4	0.046
*Σ*ω*-3*	13.3 ± 3.8	8.9 ± 2.3	0.009	12.8 ± 3.9	7.7 ± 3.1	0.028
*18 : 3*ω*-3 *(ALA)	0.06 ± 0.05	0.10 ± 0.05	0.059	0.08 ± 0.06	0.11 ± 0.09	ns
*20 : 5*ω*-3 *(EPA)	4.6 ± 1.3	2.3 ± 0.7	0.005	4.8 ± 0.8	2.3 ± 0.9	0.028
*22 : 6*ω*-3 *(DHA)	7.5 ± 2.2	5.5 ± 1.5	0.022	6.7 ± 3.1	4.3 ± 2.2	0.028
**ω*-6/*ω*-3 *ratio	3.1 ± 0.9	5.3 ± 1.6	0.005	3.4 ± 1.3	6.6 ± 2.9	0.028

PS						
*Σ*ω*-6*	13.8 ± 3.1	17.3 ± 2.5	0.008	14.1 ± 4.4	19.0 ± 6.7	0.028
*18 : 2*ω*-6 *(LA)	7.3 ± 2.0	9.17 ± 1.8	0.021	7.2 ± 4.2	10.9 ± 6.3	0.028
*20 : 4*ω*-6 *(AA)	4.1 ± 1.1	5.7 ± 1.4	0.021	4.1 ± 1.1	5.7 ± 2.4	0.018
*Σ*ω*-3*	18.5 ± 3.1	16.4 ± 4.0	ns	20.1 ± 6.9	19.1 ± 6.4	ns
*18 : 3*ω*-3 *(ALA)	0.01 ± 0.05	0.04 ± 0.12	ns	0.02 ± 0.06	0.21 ± 0.42	ns
*20 : 5*ω*-3 *(EPA)	0.3 ± 0.3	0.1 ± 0.2	0.075	0.3 ± 0.2	0.2 ± 0.1	0.028
*22 : 6*ω*-3 *(DHA)	13.6 ± 2.6	12.2 ± 3.5	ns	15.1 ± 6.1	14.1 ± 5.8	ns
**ω*-6/*ω*-3 *ratio	0.8 ± 0.2	1.1 ± 0.4	0.015	0.8 ± 0.5	1.2 ± 0.8	0.028

PI						
Σ*ω*-6*	46.1 ± 1.7	45.7 ± 1.8	ns	45.2 ± 0.8	46.6 ± 1.0	0.046
*18 : 2*ω*-6 *(LA)	3.9 ± 0.8	4.1 ± 0.8	ns	3.0 ± 1.8	3.7 ± 1.7	ns
*20 : 4*ω*-6 *(AA)**	34.1 ± 2.3	32.6 ± 2.3	0.066	33.0 ± 3.4	34.0 ± 3.6	0.028
*Σ*ω*-3*	1.6 ± 0.5	1.5 ± 1.0	ns	2.2 ± 1.1	1.7 ± 0.9	ns
*18 : 3*ω*-3 *(ALA)	<0.01	<0.01		<0.01	<0.01	
*20 : 5*ω*-3 *(EPA)	0.09 ± 0.09	0.02 ± 0.03	0.042	0.08 ± 0.07	0.03 ± 0.04	0.068
*22 : 6*ω*-3 *(DHA)	0.7 ± 0.3	0.8 ± 0.8	ns	0.9 ± 0.9	0.8 ± 0.6	ns
**ω*-6/*ω*-3 *ratio	31.1 ± 9.1	47.5 ± 35.5	0.086	26.4 ± 14.3	34.3 ± 18.4	*ns*

DPG						
*Σ*ω*-6*	92.0 ± 4.9	90.4 ± 2.2	ns	89.9 ± 3.2	91.9 ± 2.0	ns
*18 : 2*ω*-6 *(LA)	90.8 ± 4.9	89.0 ± 2.1	ns	87.5 ± 3.7	89.9 ± 2.4	0.028
*20 : 4*ω*-6 *(AA)	0.7 ± 0.4	0.5 ± 1.0	ns	1.7 ± 0.7	1.3 ± 0.5	ns
*Σ*ω*-3*	1.1 ± 0.4	1.1 ± 1.0	ns	1.7 ± 0.5	1.2 ± 0.4	0.063
*18 : 3*ω*-3 *(ALA)	0.6 ± 0.2	0.5 ± 0.2	ns	0.5 ± 0.1	0.6 ± 0.1	0.091
*20 : 5*ω*-3 *(EPA)	0.13 ± 0.18	0.05 ± 0.07	0.075	0.34 ± 0.16	0.12 ± 0.08	0.028
*22 : 6*ω*-3 *(DHA)	0.2 ± 0.2	0.5 ± 1.0	ns	0.5 ± 0.3	0.2 ± 0.3	0.046
**ω*-6/*ω*-3 *ratio	94.2 ± 35.5	120.4 ± 85.8	ns	56.6 ± 19.4	81.7 ± 20.0	0.063

Data are presented as means ± SD. *P* values of paired Wilcoxon signed-ranks test of the values after *n*-3 and placebo treatment. *P < 0.05, **P < 0.01, comparing males and females by delta values.
